# Comparative Outcomes Between Black and White Patients With Early‐Stage Diffuse Large B‐Cell Lymphoma

**DOI:** 10.1002/cam4.71680

**Published:** 2026-03-03

**Authors:** María Herrán, Sindu Iska, Hong Liang, Ludovic Saba, Chakra P. Chaulagain, Chieh‐Lin Fu

**Affiliations:** ^1^ Department of Hematology and Oncology, Maroone Cancer Center Cleveland Clinic Florida Weston Florida USA; ^2^ Hematology Oncology Medical College of Georgia Augusta Georgia; ^3^ Department of Clinical Research Cleveland Clinic Florida Weston Florida USA; ^4^ Internal Medicine University of Texas Southwestern Medical Center Dallas Texas USA

**Keywords:** early‐stage diffuse large B‐cell lymphoma, health disparities, survival outcomes

## Abstract

**Introduction:**

Determinants of survival in DLBCL stage I–IV collectively have reported Black patients and advanced stage to have worse outcomes on multivariate analysis. As advanced stage is a factor in outcome, available data have not focused on early‐stage DLBCL to evaluate the impact of race and SES on outcome. To address this research gap, the study aims to examine and compare the characteristics and the determinants of survival between US Black and White patients with early‐stage (I–II) DLBCL.

**Methods:**

A retrospective NCDB analysis of early‐stage DLBCL patients was conducted, comparing sociodemographic, clinical, and treatment factors between Black and White groups. Chi‐square tests assessed differences. Multivariate Cox regression identified survival predictors. Matched patient pairs were analyzed using Cox models with robust variance, and Kaplan–Meier curves evaluated survival outcomes by race.

**Results:**

81,430 early‐stage DLBCL patients diagnosed between 2004 and 2017 included 5,709 Black and 75,721 White patients. Multivariate analysis showed higher mortality risk for males (HR 1.09), aged ≥ 60 (HR 2.04), ≥ 2 comorbidities (HR 1.92), B symptoms (HR 1.23), HIV infection (HR 1.59), and lower income (HR 1.18) (all *p* < 0.0001). Notably, survival rates between Black and White patients were not significantly different (HR 0.98, *p* = 0.5263).

**Conclusion:**

Black patients have been reported to have worse survival for DLBCL, considering all stages collectively. The remarkable finding of our study was that despite disparities in socioclinical determinants, no overall survival difference was observed between Black and White patients with early‐stage DLBCL. We suggest that the similar survival in early‐stage DLBCL for Black and White patients is primarily overcome by the favorable remission rate of early‐stage DLBCL and accessibility to conventional chemoimmunotherapy. We also imply that the survival disparity between Black and White patients in DLBCL is more likely to be the impact of socioclinical determinants on advanced stage III/IV DLBCL.

## 1 Introduction

Diffuse Large B‐Cell Lymphoma (DLBCL) is the most common type of non‐Hodgkin lymphoma (NHL), accounting for approximately 30% of cases [1, 2]. In the United States (US), DLBCL exhibits racial variation, with White Americans having higher incidence rates compared to Black, Asian, and American Indian or Alaska Native individuals, in decreasing order of occurrence. DLBCL tends to occur more frequently in males, accounting for approximately 55% of cases [2]. Around two‐thirds of patients with DLBCL are diagnosed with advanced‐stage (III/IV) disease. Early‐stage (I/II) DLBCL has a favorable 10‐year overall survival (OS) rate of at least 80% [3, 4, 5]. Utilizing rituximab‐based chemotherapy as a curative treatment approach for all stages of DLBCL has demonstrated long‐term survival in over two‐thirds of patients and is currently the standard of care [3, 4, 5]. Despite advances in the treatment of DLBCL, determinants of survival in DLBCL stage I–IV have collectively reported Black patients and advanced stage (III–IV) to have worse outcomes on multivariate analysis [6, 7, 8, 9, 10]. Black patients with DLBCL have a risk of death that is 10% to 20% higher than the non‐Hispanic White population, noted since rituximab approval [6, 11]. Several studies suggest an association of race with socioeconomic status (SES), which was shown to affect DLBCL mortality and potential access to care [12, 13, 14, 15]. As advanced stage is a factor in outcome, available data have not focused, however, on early‐stage DLBCL to evaluate the impact of race and SES on outcome as reported. To address this research gap, the present study aims to examine and compare the characteristics and the determinants of survival between US Black and White patients diagnosed with early‐stage (I–II) DLBCL to identify opportunities for interventions for a highly curable subgroup.

## 2 Materials and Methods

### 2.1 Data Source

We aimed to determine the characteristics and outcomes for stage I and II DLBCL between Black and White patients using a retrospective analysis obtained from the National Cancer Database (NCDB) from 2004 to 2017. The NCDB is a comprehensive hospital‐based oncology database that captures around 70% of newly diagnosed cancer cases across over 1500 medical facilities in the United States, including those in Puerto Rico [16]. The NCDB is supported by the American College of Surgeons and the American Cancer Society. Since the NCDB contains de‐identified patient data, the study was exempt from Institutional Review Board approval. This study followed the Strengthening Reporting of Observational Studies in Epidemiology (STROBE) guidelines. More detailed information on available treatments and outcomes is available in the NCDB than in the Surveillance, Epidemiology, and End Results (SEER) database. The NCDB includes the use of chemoimmunotherapy, comorbidities, primary payer, facility characteristics, and other measures of sociomedical determinants. Fewer DLBCL patients in the NCDB cohort had an unknown stage compared to those in the SEER cohort, as reported in one publication [7].

### 2.2 Patient Selection

Inclusion criteria comprised the following: (1) aged at least 18 years; (2) diagnosed with early‐stage (I or II) DLBCL from 2004 to 2017. Exclusion criteria included patients with unknown or unreported data regarding race or ethnicity, stage, chemotherapy, hormone therapy, and immunotherapy.

### 2.3 Clinical Variables

Sociodemographic data (age, race, ethnicity, gender, median household income, insurance status, education, facility type, distance to treatment facility, and facility location) and clinical characteristics (comorbidities, year of diagnosis, stage, HIV infection, presence of B symptoms, chemotherapy, hormone therapy, and immunotherapy) were included in the analysis. The standard treatment for early‐stage DLBCL is chemoimmunotherapy, conventionally cyclophosphamide, doxorubicin, vincristine, prednisone with rituximab (CHOP‐R) [16]. Approved in 1997, rituximab is a CD20 monoclonal antibody reported as an immunotherapy, while prednisone is reported as hormone therapy in the NCDB treatment categories.

### 2.4 Statistical Analysis

Descriptive statistics were used to summarize the patients' characteristic variables. The Chi‐square test and the Wilcoxon rank sum test were used to evaluate the differences in characteristic variables between White and Black populations. Multivariate Cox regression analysis with backward elimination was used to identify independent predictors of survival. Additionally, a Kaplan–Meier survival curve was generated for the patient cohort. Furthermore, a White and Black group‐matched sample was constructed by exact matching for all characteristic variables, and the Cox proportional hazards model with robust variance estimators was used to compare the survivorships between the White and Black patients in the matched pairs. SAS version 9.4 was used to analyze the data.

## 3 Results

**FIGURE 1:**
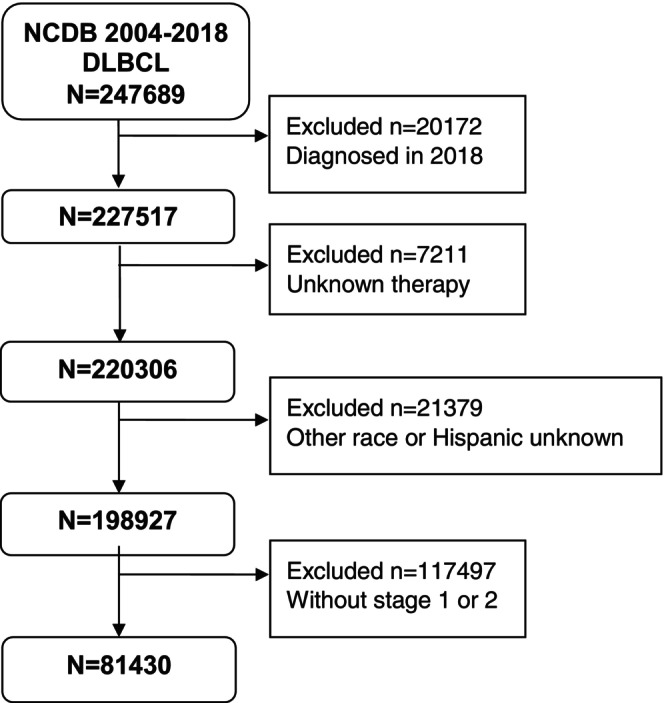
Consort Diagram.

**FIGURE 2:**
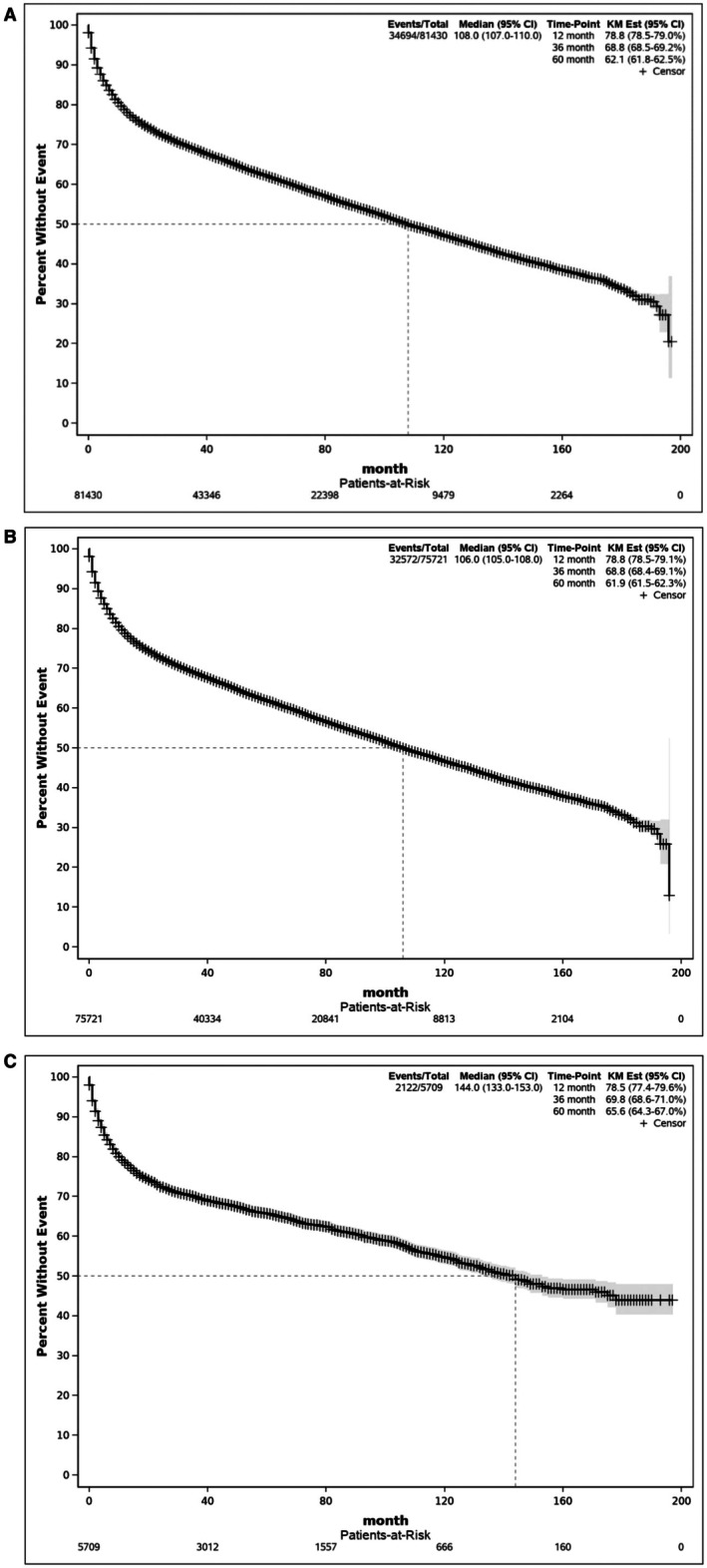
A. Overall Kaplan–Meier survival curve for the whole cohort. Figure 2B. Overall Kaplan–Meier survival curve for White patients. Figure 2C. Overall Kaplan–Meier survival curve for Black patients.

81,430 deidentified patients diagnosed with early‐stage DLBCL between 2004 and 2017 were available for evaluation (Figure 1); 5709 (7.0%) were Black and 75,721 (93%) were White. Detailed clinical and demographic characteristics of White and Black patients are outlined in Table 1. In this dataset of early‐stage DLBCL, multivariable Cox regression analysis using the backward elimination method revealed multiple factors associated with increased mortality, including age, gender, ethnicity, income, insurance, distance traveled, Charlson‐Deyo Comorbidity Index, stage, HIV infection, B symptoms, and types of treatment received (Table 2). Higher mortality risk was observed in males (HR 1.09, 95% CI 1.06–1.11, *p* < 0.0001). As expected, higher risk of death was observed in patients 60 years or older (HR 2.04, 95% CI 1.96–2.12, *p* < 0.0001); with ≥ 2 comorbidities (HR 1.92, 95% CI 1.85–1.98, *p* < 0.0001); presenting with B‐symptoms (HR 1.23, 95% CI 0.53–0.68, *p* < 0.0001), HIV infection (HR 1.59, 95% CI 1.48–1.70) and lower household income (HR 1.18, 95% CI 1.11–1.18, *p* < 0.0001). Interestingly, Hispanic patients had decreased risk of death compared to non‐Hispanic patients (HR 0.84, 95% CI 0.80–0.89, *p* < 0.0001). Notably, survival rates between Black and White patients were not significantly different (HR 0.98, 95% CI 0.94–1.03, *p* = 0.5263). Kaplan–Meier analyses showed that whole cohort survival rates at 1‐, 3‐, or 5‐year were 78.8%, 68.8%, and 62.1%, respectively (Figure 2A). Kaplan–Meier survival rates for White patients at 1‐, 3‐, or 5‐year were 78.8%, 68.8%, and 61.9%, respectively (Figure 2B). Kaplan–Meier survival rates for Black patients at 1‐, 3‐, or 5‐year were 78.5%, 69.8%, and 65.6%, respectively (Figure 2C).

**TABLE 1:** Patient clinical characteristics.

Variable	All Patients			Case‐Matched Patients	
	White (*n* = 75,721)	Black (*n* = 5,709)	*p*‐value	White (*n* = 1,253)	Black (*n* = 1,253)
Age, median (IQR)	68.0 (56.0–78.0)	56.0 (43.0–68.0)	< 0.0001		
Age					
Male, *n* (%)	40,703 (53.0)	2,949 (51.7)	0.0022	611 (48.8)	611 (48.8)
Hispanic, *n* (%)	5,596 (7.4)	76 (1.3)	0.0022	4 (0.32)	4 (0.32)
Education					
< 7.0%	19,006 (25.1)	530 (9.3)	< 0.0001	210 (16.8)	210 (16.8)
7.0%–12.9%	24,068 (31.8)	1,132 (19.8)		369 (29.4)	369 (29.4)
13.0%–20.9%	16,484 (21.8)	1,824 (31.9)		390 (31.1)	390 (31.1)
> 21.0%	10,104 (13.3)	1,722 (30.2)		284 (22.7)	284 (22.7)
NA	6,063 (8.0)	501 (8.8)			
Median household income, *n* (%)			0.0001		
< 38,000	9,478 (12.5)	2,040 (35.7)		358 (28.6)	358 (28.6)
38,000–47,000	16,181 (21.4)	1,182 (20.7)		264 (21.1)	264 (29.1)
48,000–62,000	19,265 (25.4)	1,059 (18.6)		285 (22.7)	285 (22.7)
> 63,000	24,697 (32.6)	923 (16.2)		346 (27.6)	346 (27.6)
NA	6,100 (8.1)	505 (8.8)			
Insurance status, *n* (%)					
Private	27,672 (36.5)	2,252 (39.4)	< 0.0001	547 (43.7)	547 (43.7)
Medicare	40,339 (53.3)	1,884 (33.0)		645 (51.5)	645 (51.5)
Medicaid/Other	4,191 (5.5)	979 (17.2)		6 (0.5)	6 (0.5)
Not‐insured	2,164 (2.9)	447 (7.8)		17 (1.3)	17 (1.3)
Unknown	1,355 (1.8)	147 (2.6)		38 (3.0)	38 (3.0)
Year of diagnosis, *n* (%)			0.0527		
2004–2007	17,411 (23.0)	1,257 (22.0)		252 (20.1)	252 (20.1)
2008–2011	20,885 (27.6)	1,539 (27.0)		420 (33.5)	420 (33.5)
2012–2014	18,162 (24.0)	1,453 (25.4)		254 (20.3)	254 (20.3)
2015–2017	19,263 (25.4)	1,460 (25.6)		327 (26.1)	327 (26.1)
Charlson‐Deyo Score, *n* (%)					
0	56,847 (75.1)	4,076 (71.4)	< 0.0001	1,077 (86.0)	1,077 (86.0)
1	12,491 (16.5)	873 (15.3)		129 (10.3)	129 (10.3)
≥ 2	6,383 (8.4)	760 (13.3)		47 (3.7)	47 (3.7)
Stage, *n* (%)			< 0.0001		
1	43,962 (58.1)	3,100 (54.3)		731 (58.3)	731 (58.3)
2	31,759 (41.9)	2,609 (45.7)		522 (41.7)	522 (41.7)
HIV, *n* (%)					
Positive	1,304 (1.7)	753 (13.2)	< 0.0001	10 (0.8)	10 (0.8)
Negative	43,177 (57.0)	3,170 (55.5)		847 (67.6)	847 (67.6)
NA	31,240 (41.3)	1,786 (31.3)		396 (31.6)	396 (31.6)
B symptoms, *n* (%)			< 0.0001		
B symptoms	13,313 (17.6)	1,402 (24.6)		147 (11.7)	147 (11.7)
No B symptoms	56,634 (70.8)	3,577 (62.6)		1,025 (81.8)	1,025 (81.8)
Unknown	8,774 (11.6)	730 (12.8)		81 (6.5)	81 (6.5)
Chemotherapy, *n* (%)			0.0026		
Multi‐agent	54,816 (72.4)	4,047 (70.9)		980 (78.2)	980 (78.2)
Single/unknown agent	6,872 (9.1)	499 (8.7)		47 (3.8)	47 (3.8)
Not administered	14,033 (18.5)	1,163 (20.4)		226 (18.0)	226 (18.0)
Hormone therapy, *n* (%)	33,438 (44.2)	2,300 (40.3)	< 0.0001	565 (45.1)	565 (45.1)
Immunotherapy, *n* (%)	22,893 (30.2)	1,604 (28.1)	0.0007	359 (28.7)	359 (28.7)

**TABLE 2:** Multivariate cox analysis for survival.

Variable	HR (95% CI)	*p*‐value
Age (≥ 60 vs. < 60)	2.04 (1.96–2.12)	< 0.0001
Gender (male vs. female)	1.09 (1.06–1.11)	< 0.0001
Hispanic (yes vs. no)	0.84 (0.80–0.89)	< 0.0001
Median household income		
< 38,000	1.18 (1.14–1.22)	< 0.0001
38,000 – 47,000	1.14 (1.11–1.18)	< 0.0001
48,000 – 62,000	1.13 (1.10–1.16)	< 0.0001
> 63,000 (Ref.)	1	—
Unknown	1.25 (0.95–1.64)	0.1174
Insurance		
Private (Ref.)	1	—
Medicare	1.79 (1.73–1.84)	< 0.0001
Medicaid	1.55 (1.46–1.63)	< 0.0001
Uninsured	1.31 (1.21–1.42)	< 0.0001
Unknown	1.34 (1.23–1.46)	< 0.0001
Year of diagnosis		
2004–2007	0.99 (0.95–1.03)	0.5984
2008–2011	0.95 (0.91–0.99)	0.0102
2012–2014	1.01 (0.97–1.05)	0.6187
2015–2017 (Ref.)	1	—
Distance traveled (miles)		
< 10 (Ref.)	1	
10–23	0.93 (0.90–0.96)	< 0.0001
> 23	0.96 (0.94–0.99)	0.0059
Facility type		
Academic (Ref.)	1	—
Non‐academic	1.00 (0.98–1.03)	0.9829
Unknown	0.52 (0.48–0.56)	< 0.0001
CDC		
0 (Ref.)	1	—
1	1.37 (1.33–1.41)	< 0.0001
≥ 2	1.92 (1.85–1.98)	< 0.0001
Stage		
1 (Ref.)	1	
2	1.16 (1.13–1.18)	< 0.0001
HIV		
Negative	1	
Positive	1.59 (1.48–1.70)	< 0.0001
Unknown	1.03 (1.01–1.05)	0.0093
B symptoms		
No B symptoms (Ref.)	1	
B symptoms	1.23 (1.20–1.27)	< 0.0001
Unknown	1.00 (0.97–1.04)	0.9115
Chemotherapy		
Unadministered (Ref.)	1	
Multiagent	0.38 (0.37–0.39)	< 0.0001
Single agent	0.66 (0.63–0.68)	< 0.0001
Hormone therapy (yes vs. no)	0.86 (0.84–0.89)	< 0.0001
Immunotherapy (yes vs. no)	0.81 (0.78–0.84)	< 0.0001

Evaluation of the sociomedical variables between White and Black patients in early‐stage DLBCL revealed the following. Among the White patients, the median age was 68 years (IQR 56.0–78.0), and 56 years (IQR 43.0–68.0) for Black patients. There was an increased frequency of HIV infection, B symptoms, and a comorbidity index > 2 for Black patients compared to White patients. There were statistically significant differences in education, socioeconomic status, and type of medical coverage between the Black and White populations. Statistical differences were noted between Black and White patients in receiving chemotherapy treatment and in receiving immunotherapy, likely rituximab. Both types of treatment affect survival on multivariate analysis; immunotherapy received (HR 0.81, 95% CI 0.78–0.84) and multiagent chemotherapy received (HR 0.38, 95% CI 0.37–0.39, *p* < 0.0001). For the exact matching sample (*n* = 1,253), using all the variables in Table 1 in a 1:1 ratio, there was no significant difference in survival between White and Black groups in early‐stage DLBCL (*p* = 0.1818).

There was no significant difference in survival based on the year of diagnosis evaluated in 4‐year intervals. The frequency of diagnosis of early‐stage DLBCL between Black and White patients measured in 4‐year intervals was not different. Treatment has not changed significantly since the approval of rituximab in 1997. Therefore, outcomes measured from 2004 to 2017 are representative of practice patterns over the timespan. Our study reported a lower survival rate at 5 years for early‐stage lymphoma, as it includes a subset of untreated patients.

## 4 Discussion

In this analysis using a large nationwide cancer registry via multivariate model, we compared survival and described multiple socio‐demographic (i.e., insurance, household income, education, distance to center) and clinical factors (i.e., age, gender, Hispanic ethnicity, comorbidity, B symptoms and HIV infection) with treatment modalities associated with mortality in early‐stage (I, II) DLBCL (Table 2). Additionally, we evaluated the sociomedical determinants between Black and White patients with early‐stage DLBCL (Table 1). No significant difference in OS was found between White and Black patients in early‐stage DLBCL despite sociomedical disparities. After matching White and Black patients for homogenous characteristics, 5‐year survival remained similar. Interestingly, Hispanic patients exhibited higher survival compared to non‐Hispanic patients in our study.

Although racial disparity in survival has been observed collectively (stages I–IV) for DLBCL, we report no racial disparity in survival in early (stage I–II) DLBCL, despite finding similar disparities in sociomedical determinants as reported collectively for DLBCL. Additionally, there was no difference in survival on treatment at an academic or nonacademic center. Therefore, we suggest that the similar survival observed in early‐stage DLBCL for Black and White patients in our study is primarily overcome by the favorable remission rate of early‐stage DLBCL with chemoimmunotherapy with minimal morbidity and accessibility of a common standard therapy (CHOP‐R) that can be given at any facility. We also imply that the survival disparity between Black and White patients in DLBCL reported in published data is more likely to be the impact of socioclinical determinants on advanced stage III/IV DLBCL. We recommend that studies evaluating DLBCL outcomes across different racial groups should be stratified by stage to better determine the effect of socioclinical factors. Therefore, to narrow the survival disparity gap reported between Black and White groups, the focus should be primarily concentrated on advanced‐stage DLBCL, including access to novel treatment via clinical trials, for which distance to center and medical coverage can be barriers. We also submit that access to healthcare to detect DLBCL in the early stage, which is highly curative, may decrease the racial disparity seen across DLBCL, and therefore, awareness and education of DLBCL are warranted. Our study notes that Black patients with early‐stage DLBCL have a higher frequency of Medicaid coverage. Therefore, Medicaid expansion may have a positive impact on earlier diagnosis and treatment.

We suggest the principal factor in outcome for early‐stage DLBCL is access to standard therapy that provides excellent long‐term remission. Gene expression profiling has suggested the ABC‐subtype, associated with worse OS even after being treated with rituximab CHOP, is more commonly found in those with African American ancestry [17, 18, 19]. This would suggest that the biologic features in DLBCL in patients of African origin may not significantly influence early‐stage DLBCL. Although comorbidities and HIV infection have been borne out as variables affecting survival in early‐stage DLBCL, the differences did not appear to impact survival in early‐stage DLBCL between Black and White patients. The median age at presentation has been reported to be younger for Black Americans compared to White Americans [2, 6, 17, 18, 20, 21] for DLBCL collectively (stage I–IV). Our study of early‐stage DLBCL observed a similar age disparity.

It was interesting to note that Hispanics had a better OS in limited‐stage DLBCL, confirming previous studies that suggest better OS in Hispanics across all stages. Further studies are warranted. Blansky et al. demonstrated that Hispanics diagnosed with DLBCL had a 52% lower risk of mortality compared to non‐Hispanic whites after controlling for clinical prognostic factors [22]. It is noteworthy that Hispanics represent a broad group with different countries of origin and different genetic distributions of European, African, and Indigenous ancestry. Therefore, their unique genetic variations may contribute to their survival outcomes, and accordingly, some authors have suggested that certain genetic subtypes might have a better response to rituximab [23]. In addition, the “health immigrant effect” has been described as a condition in which those in good health are more prone to leave their country [24], and therefore, this might partly explain the results.

## 5 Strengths and Limitations

A key strength of our study is that we analyzed the largest cohort of patients diagnosed with early‐stage DLBCL. Therefore, our analysis captured a significant cohort of racial and ethnic groups, specifically Blacks and Hispanics living in the United States, providing real‐world insights. Important limitations of our analysis relate to the retrospective observational study design, as well as the nature of the database, due to the unavailability of some relevant data that would have allowed for capturing more patients, and it does not account for non‐observable confounding variables. Although the exact type of therapy is unknown, CHOP‐R remains the most common treatment. The number of cycles, time to treatment, and biologic features that may be significant in DLBCL cannot be further determined from the NCDB registry.

## 6 Conclusion

Black patients have been reported to have worse survival for DLBCL, considering both early and advanced stages collectively. However, we present the only study limited to early‐stage DLBCL that examines the survival determinants and characteristics between Black and White patients. The most remarkable finding of our study was that despite disparities in socioclinical determinants, no overall survival difference was observed between Black and White patients with early‐stage DLBCL. Our study's independent characteristics, evaluated through multivariate analysis, that were associated with a worse outcome for early‐stage DLBCL, support similar sociomedical determinants for DLBCL stages I–IV, except for race. Racial disparities and access to appropriate treatments may be more pronounced in advanced DLBCL.

Additionally, the exact matching of variables, including demographic and clinical determinants, confirmed that survival remained similar, not better. Therefore, in early‐stage DLBCL, the accessibility to standard of care with favorable remission rates and minimal morbidity, even with Medicaid being more frequent in Black patients, may be more predictive of outcome between Black and White patients and can overcome other socioclinical disparities. DLBCL should be stratified by stage to evaluate socioclinical determinants on survival. Advanced stage is a known independent prognostic feature for DLBCL. Achieving health equity in DLBCL with observed racial disparity may be more impactful on the advanced stage than for the limited stage, as there is no survival disparity between White and Black patients in stage I/II DLBCL despite the disparity in demographic and social determinants. Additionally, the conventional therapy for early DLBCL has not changed significantly and remains very favorable, for which diagnosis and treatment of DLBCL at an earlier stage (I–II) via access to healthcare may narrow the racial disparity gap observed in DLBCL.

## Author Contributions

M.H., S.I., L.S., C.P.C., and C.‐L.F. conceived and designed the study. H.L. performed statistical analysis. All authors contributed to data interpretation, participated in manuscript writing, approved the manuscript for publication, and are accountable for all aspects of the work.

## Ethics Statement

This study used de‐identified data from the National Cancer Database (NCDB), which is HIPAA‐compliant and does not contain patient identifiers. As such, the project was exempt from IRB review. All data use agreements were followed.

## Conflicts of Interest

The authors declare no conflicts of interest.

## Data Availability

The data that support the findings of this study are available from the American College of Surgeons and the American Cancer Society. Still, restrictions apply to the availability of these data, which were used under license for the current study, and so are not publicly available. Data are, however, available from the authors upon reasonable request and with permission of the American College of Surgeons and American Cancer Society.

## References

[1] S. H. Swerdlow , E. Campo , S. A. Pileri , et al., “The 2016 Revision of the World Health Organization Classification of Lymphoid Neoplasms,” Blood 127, no. 20 (2016): 2375–2390. 26980727 10.1182/blood-2016-01-643569 PMC4874220

[2] L. M. Morton , S. S. Wang , S. S. Devesa , P. Hartge , D. D. Weisenburger , and M. S. Linet , “Lymphoma Incidence Patterns by WHO Subtype in the United States, 1992–2001,” Blood 107, no. 1 (2006): 265–276. 16150940 10.1182/blood-2005-06-2508 PMC1895348

[3] S. Susanibar‐Adaniya and S. K. Barta , “2021 Update on Diffuse Large B Cell Lymphoma: A Review of Current Data and Potential Applications on Risk Stratification and Management,” American Journal of Hematology 96, no. 5 (2021): 617–629. 33661537 10.1002/ajh.26151 PMC8172085

[4] E. A. Hawkes , A. Barraclough , and L. H. Sehn , “Limited‐Stage Diffuse Large B‐Cell Lymphoma,” Blood 139, no. 6 (2022): 822–834. 34932795 10.1182/blood.2021013998

[5] A. E. Rojek and S. M. Smith , “Evolution of Therapy for Limited Stage Diffuse Large B‐Cell Lymphoma,” Blood Cancer Journal 12, no. 2 (2022): 33. 35210407 10.1038/s41408-021-00596-z PMC8867133

[6] A. A. Phillips and D. A. Smith , “Health Disparities and the Global Landscape of Lymphoma Care Today,” in American Society of Clinical Oncology Educational Book, vol. 37 (Wolters Kluwer Health, Inc, 2017), 526–534. 28561692 10.1200/EDBK_175444

[7] C. R. Flowers , S. A. Fedewa , A. Y. Chen , et al., “Disparities in the Early Adoption of Chemoimmunotherapy for Diffuse Large B‐Cell Lymphoma in the United States,” Cancer Epidemiology, Biomarkers & Prevention 21, no. 9 (2012): 1520–1530. 10.1158/1055-9965.EPI-12-0466 PMC4155492 22771484

[8] K. MacDougall , S. Day , S. Hall , et al., “Impact of Race and Age and Their Interaction on Survival Outcomes in Patients With Diffuse Large B‐Cell Lymphoma,” Clinical Lymphoma, Myeloma & Leukemia 23, no. 5 (2023): 379–384. 10.1016/j.clml.2023.01.015 36813625

[9] P. Dhakal , B. Chen , S. Giri , J. M. Vose , J. O. Armitage , and V. R. Bhatt , “Effects of Center Type and Socioeconomic Factors on Early Mortality and Overall Survival of Diffuse Large B‐Cell Lymphoma,” Future Oncology 15, no. 18 (2019): 2113–2124. 31144521 10.2217/fon-2018-0596

[10] M. Wang , K. D. Burau , S. Fang , H. Wang , and X. L. Du , “Ethnic Variations in Diagnosis, Treatment, Socioeconomic Status, and Survival in a Large Population‐Based Cohort of Elderly Patients With Non‐Hodgkin Lymphoma,” Cancer 113, no. 11 (2008): 3231–3241. 18937267 10.1002/cncr.23914 PMC6296219

[11] C. R. Flowers , P. J. Shenoy , U. Borate , et al., “Examining Racial Differences in Diffuse Large B‐Cell Lymphoma Presentation and Survival,” Leukemia & Lymphoma 54, no. 2 (2013): 268–276. 22800091 10.3109/10428194.2012.708751 PMC4151307

[12] R. S. Komrokji , N. H. Al Ali , M. S. Beg , et al., “Outcome of Diffuse Large B‐Cell Lymphoma in the United States Has Improved Over Time but Racial Disparities Remain: Review of SEER Data,” Clinical Lymphoma, Myeloma & Leukemia 11, no. 3 (2011): 257–260. 10.1016/j.clml.2011.03.012 21658652

[13] R. Griffiths , M. Gleeson , K. Knopf , and M. Danese , “Racial Differences in Treatment and Survival in Older Patients With Diffuse Large B‐Cell Lymphoma (DLBCL),” BMC Cancer 10 (2010): 625. 21073707 10.1186/1471-2407-10-625 PMC2995801

[14] S. B. Aqeel , M. S. Faisal , O. S. Akhtar , et al., “Racial and Ethnic Disparities in Outcomes of Diffuse Large B Cell Lymphoma in Adolescent and Young Adults: A SEER Database Analysis,” Annals of Hematology 103, no. 12 (2024): 5539–5547. 39495284 10.1007/s00277-024-06075-2

[15] L. Tao , J. M. Foran , C. A. Clarke , S. L. Gomez , and T. H. M. Keegan , “Socioeconomic Disparities in Mortality After Diffuse Large B‐Cell Lymphoma in the Modern Treatment Era,” Blood 123, no. 23 (2014): 3553–3562. 24705494 10.1182/blood-2013-07-517110 PMC4047495

[16] K. Y. Bilimoria , A. K. Stewart , D. P. Winchester , and C. Y. Ko , “The National Cancer Data Base: A Powerful Initiative to Improve Cancer Care in the United States,” Annals of Surgical Oncology 15, no. 3 (2008): 683–690. 18183467 10.1245/s10434-007-9747-3 PMC2234447

[17] G. S. Nowakowski and M. S. Czuczman , “ABC, GCB, and Double‐Hit Diffuse Large B‐Cell Lymphoma: Does Subtype Make a Difference in Therapy Selection?,” American Society of Clinical Oncology Educational Book 35 (2015): e449–e457. 10.14694/EdBook_AM.2015.35.e449 25993209

[18] M. J. Lee , J. L. Koff , J. M. Switchenko , et al., “Genome‐Defined African Ancestry Is Associated With Distinct Mutations and Worse Survival in Patients With Diffuse Large B‐Cell Lymphoma,” Cancer 126, no. 15 (2020): 3493–3503. 32469082 10.1002/cncr.32866 PMC7494053

[19] L. J. Nastoupil , R. Pauly , L. Bernal‐Mizrachi , et al., “Exome Sequencing of African American Siblings With Activated, B‐Cell Like Diffuse Large B‐Cell Lymphoma,” Blood 122, no. 21 (2013): 3014–3014.

[20] P. J. Shenoy , N. Malik , R. Sinha , et al., “Racial Differences in the Presentation and Outcomes of Chronic Lymphocytic Leukemia and Variants in the United States,” Clinical Lymphoma, Myeloma & Leukemia 11, no. 6 (2011): 498–506. 10.1016/j.clml.2011.07.002 21889433

[21] S. S. Wang , “Epidemiology and Etiology of Diffuse Large B‐Cell Lymphoma,” Seminars in Hematology 60, no. 5 (2023): 255–266. 38242772 10.1053/j.seminhematol.2023.11.004 PMC10962251

[22] D. Blansky , M. Fazzari , I. Mantzaris , T. Rohan , and H. D. Hosgood , “Racial and Ethnic Differences in Diffuse Large B‐Cell Lymphoma Survival Among an Underserved, Urban Population,” Leukemia & Lymphoma 62, no. 3 (2021): 581–589. 33112182 10.1080/10428194.2020.1839656 PMC7940576

[23] R. Schmitz , G. W. Wright , D. W. Huang , et al., “Genetics and Pathogenesis of Diffuse Large B‐Cell Lymphoma,” New England Journal of Medicine 378, no. 15 (2018): 1396–1407. 29641966 10.1056/NEJMoa1801445 PMC6010183

[24] J. T. McDonald , M. Farnworth , and Z. Liu , “Cancer and the Healthy Immigrant Effect: A Statistical Analysis of Cancer Diagnosis Using a Linked Census‐Cancer Registry Administrative Database,” BMC Public Health 17, no. 1 (2017): 296. 28381211 10.1186/s12889-017-4190-2 PMC5382414

